# Research on Electric Oil–Pneumatic Active Suspension Based on Fractional-Order PID Position Control

**DOI:** 10.3390/s24051644

**Published:** 2024-03-02

**Authors:** Yaozeng Hu, Jianze Liu, Zhuang Wang, Jingming Zhang, Jiang Liu

**Affiliations:** Institute of Mechanical and Automotive Engineering, Qingdao University of Technology, Qingdao 266520, China; huyaozeng@qut.edu.cn (Y.H.); wangzhuang010227@163.com (Z.W.); zhangjingming@geely-sunwoda.com (J.Z.); liujiang@qut.edu.cn (J.L.)

**Keywords:** fractional-order damping, oil–pneumatic actuator, FOPID control, particle swarm algorithm, active suspension

## Abstract

In this study, an electric oil and gas actuator based on fractional-order PID position feedback control is proposed, through which the damping coefficient of the suspension system is adjusted to realize the active control of the suspension. An FOPID algorithm is used to control the motor’s rotational angle to realize the damping adjustment of the suspension system. In this process, the road roughness is collected by the sensors as the criterion of damping adjustment, and the particle swarm algorithm is utilized to find the optimal objective function under different road surface slopes, to obtain the optimal cornering value. According to the mathematical and physical model of the suspension system, the simulation model and the corresponding test platform of this type of suspension system are built. The simulation and experimental results show that the simulation results of the fractional-order nonlinear suspension model are closer to the actual experimental values than those of the traditional linear suspension model, and the accuracy of each performance index is improved by more than 18.5%. The designed active suspension system optimizes the body acceleration, suspension dynamic deflection, and tire dynamic load to 89.8%, 56.7%, and 73.4% of the passive suspension, respectively. It is worth noting that, compared to traditional PID control circuits, the FOPID control circuit designed for motors has an improved control performance. This study provides an effective theoretical and empirical basis for the control and optimization of fractional-order nonlinear suspension systems.

## 1. Introduction

The current research on oil and gas suspension [1], air suspension [2], and magnetorheological suspension [3] mainly considers partial elasticity or damping characteristics, which do not reflect the memory characteristics of actual suspension damping materials. Based on the good memory function of the fractional-order model, scholars at home and abroad have proposed using fractional-order calculus theory to describe the suspension dynamics model with viscoelasticity [4]. Du et al. [5] constructed an inertia capacitive oil–air suspension device integrating ball screw inertia packages and double-cylinder oil–air springs, and by analyzing the mechanism of elasticity, damping, and inertia forces of the suspension device, they revealed the coupling relationships between the parameters revealed, and three mutually independent key parameters were extracted. By analyzing the mechanism of the elastic force and damping force, the coupling relationships between the parameters were revealed, three independent key structural parameters were extracted, and the optimal values of the three key structural parameters were determined by simulation and testing. Li et al. [6] established a multivariate exponential suspension model considering the hysteresis effect of the actual gas in the oil and gas suspension model and established a general dynamics model of the whole vehicle suspension by the vectorial method, and further deduced the equations of the stiffness and damping characteristics in different working conditions. Sun et al. [7], in order to more accurately describe the vibration characteristics of vehicles equipped with oil and gas suspension, based on the suspension characteristics of an oil and gas suspension multiphase medium, introduced the fractional calculus theory, established the fractional Bagley–Torvik equation, and used the low-pass filter of the Oustaloup algorithm to carry out numerical calculations, so as to obtain the optimal numerical solution of the nonlinear fractional-order suspension mathematical model. All of the above studies verified the applicability and validity of the fractional-order calculus theory in the modeling of automotive suspension systems and established a specific nonlinear suspension system model, but lacked experimental evidence of the reliability of the established mathematical model. Therefore, not only was the relevant model established for the studied oil and gas suspension system, but the accuracy of the model was also verified through experiments [8].

Currently, the hardware part of the field is relatively mature, and the research focuses on further enhancement of its control algorithms. In order to improve the suspension system drooping dynamics performance, Narwade et al. [9] studied the modeling and simulation of an automotive semiactive suspension system based on a PID controller and carried out the simulation study of the PID controller’s application for automotive suspensions in a more systematic way; Li et al. [10] used a genetic algorithm to tune the PID controller and the fuzzy control theory for nonlinear suspension system control to achieve multiobjective optimization of a suspension system. Liu et al. [11] proposed an adaptive neural network control scheme for active suspension with time-varying vertical displacement and velocity constraints as well as an active suspension system with an unknown body mass, and the feasibility and reasonableness of the proposed method were verified by simulation. Although the above control methods achieved a good control effect for the working conditions of a single constant, they did not consider the complex and variable conditions of the control problem. MM Kaldas et al. [12] used optimization algorithms based on the gradient algorithm to evaluate the performance of a controller in different driving conditions and a model of an active suspension controller for analytical research; their simulation studies show that the controller has an effect on the vehicle’s comfort and grip in different working conditions. Guo et al. [13] proposed an active suspension control strategy based on an inertial measurement unit as a way to compensate the body attitude and improve the ride comfort and operational stability of a vehicle on gravel and sloped roads. M J et al. [14] proposed an optimal fuzzy adaptive robust proportional–integral–derivative controller, which was designed by the gradient descent method and chain derivative rule, and utilized a particle swarm optimization algorithm to determine the optimal gain of the designed controller for the vehicle. The results show that the proposed controller is advantageous. Liang et al. [15] used a long short-term memory (LSTM) network to recognize road information, and the CDC (continuous damper) was controlled according to the road recognition information to realize adaptive damping switching control. The optimal damping coefficient was calculated after testing on different roads. While the above control algorithms can improve ride comfort and stability under complex working conditions, many excellent semiactive and active suspension schemes have also been proposed [16,17,18,19]. However, the control methods for oil–air suspension, air suspension, and magnetorheological suspension do not involve the consideration of the memory properties of suspension-damping materials. Therefore, it is necessary to study a fractional-order nonlinear suspension system containing nonlinear stiffness and fractional-order damping and to optimize the fractional-order damping coefficient parameters of this system. The main problem faced in the study of such suspension systems is the conversion of the linear damping forces in the suspension system into fractional-order damping forces [20].

You et al. [21] introduced two tuning parameters to adjust the suspension stiffness and damping and considered using fractional-order damping force to simulate the viscoelasticity of materials. The particle swarm algorithm was utilized to optimize these two tuning values. By establishing a 2-DOF fractional-order passive suspension system, the comfort and stability of the vehicle were simulated and optimized. On the other hand, Chang et al. [22] studied the active control of a fractional-order nonlinear suspension system and developed a suspension system feedback linearization method based on differential geometry. The optimal control rates were obtained using LQR control, thereby optimizing the damping performance of the suspension system.

However, the above studies only used simulations to verify the control effects. Therefore, to verify the optimization degree of the fractional-order nonlinear suspension model more comprehensively, this paper constructs a fractional-order nonlinear oil–air suspension test bench. The optimization effect of the model is verified through the comparison of simulation and experimental results. Meanwhile, for the high sensitivity and high characteristics of the fractional-order nonlinear oil–air suspension model, FOPID control with a wider range of parameter tuning is adopted in the control algorithm to realize more accurate control of the target parameters. The optimization of target parameters is based on the optimization of particle swarm optimization algorithms for different road surfaces [23].

**Figure 1:**
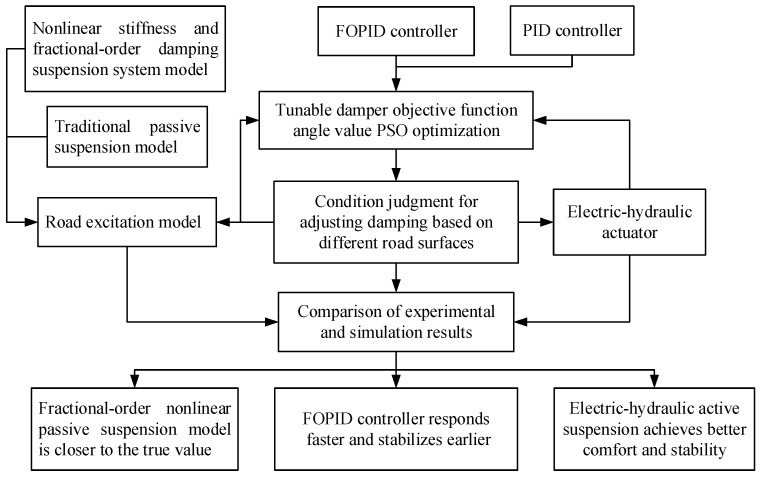
Technological routes of the paper.

To meet the comfort and stability requirements of active control with fractional-order nonlinear suspension under multiple road conditions, new fractional-order nonlinear mathematical models and matching degree accurate control algorithms are very necessary [24]. This paper consists of five parts. Section 1, Introduction, which points out some concepts and the current research status; Section 2 contains the mathematical and physical model construction of vehicle suspension system, road conditions, electric oil and gas actuators, etc.; Section 3 gives the design of the FOPID and the optimization of the objective function; and Section 4 completes the computation, simulation, and experimental implementation and discussion. Finally, some comments will be indicated in the Conclusions section. In the following sections of the article, specific details are offered. Shown in Figure 1 are the technology routes of the paper.

## 2. Principle and Modeling

### 2.1. Electric Oil–Pneumatic Active Suspension Working Principle

**Figure 2:**
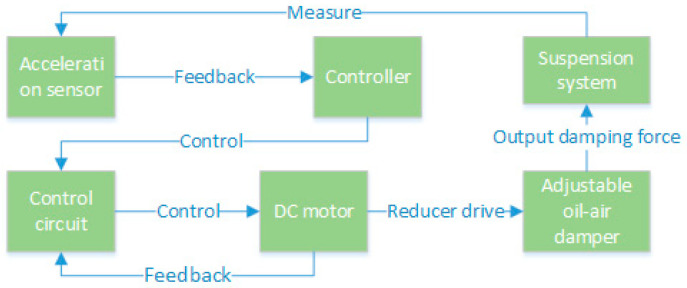
Oil–pneumatic active suspension workflow.

The design of the electric oil–air active suspension used in this paper is mainly based on an oil–air damper with adjustable damping. The suspension system measures the acceleration of the vehicle through acceleration sensors and feeds the signal back to the controller. The controller manipulates the rotation angle of the DC motor through a control circuit, which in turn realizes the damping adjustment of the damper through a gearbox transmission. This process allows the actuator to output adjustable damping force, thus realizing active control of the suspension system. The workflow of the hydro-pneumatic active suspension is shown in Figure 2.

### 2.2. Fractional-Order Damping Force Model for the Electric Oil–Pneumatic Actuator

Since the characteristics of electric oil and gas actuators are affected by a variety of factors, including conditions such as excitation frequency, external temperature, and usage history, this is consistent with the mechanical properties of viscoelastic materials. Since viscoelastic materials have both viscous and elastic properties, it is more accurate and reasonable to use a fractional-order calculus model [25] describing viscoelastic materials to depict the mechanical properties of the oil and gas damper. The fractional-order damped Duffing system model [26] describing the viscoelastic characteristics of the material is referenced in the design of the oil and gas suspension model. The fractional-order damping force at the output of the actuator can be calculated by the following equation.

(1) $$F_{c}=c_{s}\cdot D^{p}(x_{2}-x_{1}),0<p<1$$

where $x_{2}$ is the spring-loaded mass displacement, $x_{1}$ is the unsprung mass displacement, *p* is the order of the fractional-order differential term, and $c_{s}$ is the fractional-order viscoelastic damping coefficient of the suspension system. $D^{p}\left(.\right)$ is the fractional-order calculus operator, and the three main definitions of fractional-order calculus used so far are the Riemann–Liouville formula, Grunwald–Letnikov formula, and Caputo formula. In this paper, the Grunwald–Letnikov formula [27] is used to define the fractional-order calculus operator, and its differential definition is shown in the following equation.

(2) Dtpt0GLf(t)=limh→01hp∑j=0[(t−t0)/h](−1)j(pj)f(t−jh)

where $[(t-t_{0})/h]$ means taking the nearest integer to $(t-t_{0})/h$; $\left(\begin{array}{c} \alpha \\ j \end{array}\right)$ is the coefficient of the binomial; the definition of the integral only requires changing the fractional-order *p* in the differential Equation (2) to −*p*.

Since the damping of the oil–pneumatic actuator used is adjustable, the fractional-order viscoelastic damping coefficient $c_{s}$ and the actuator damping force $F_{c}$ are calculated as shown in Equations (3) and (4).

(3) $$c_{s}=b\cdot c$$

(4) $$F_{c}=b\cdot c\cdot D^{p}(x_{2}-x_{1}),0<p<1$$

where *b* is the damping scale of adjustable dampers, 10 ≥ *b* ≥ 1; *c* is the damping coefficient of the passive suspension.

### 2.3. Suspension System Modeling

**Figure 3:**
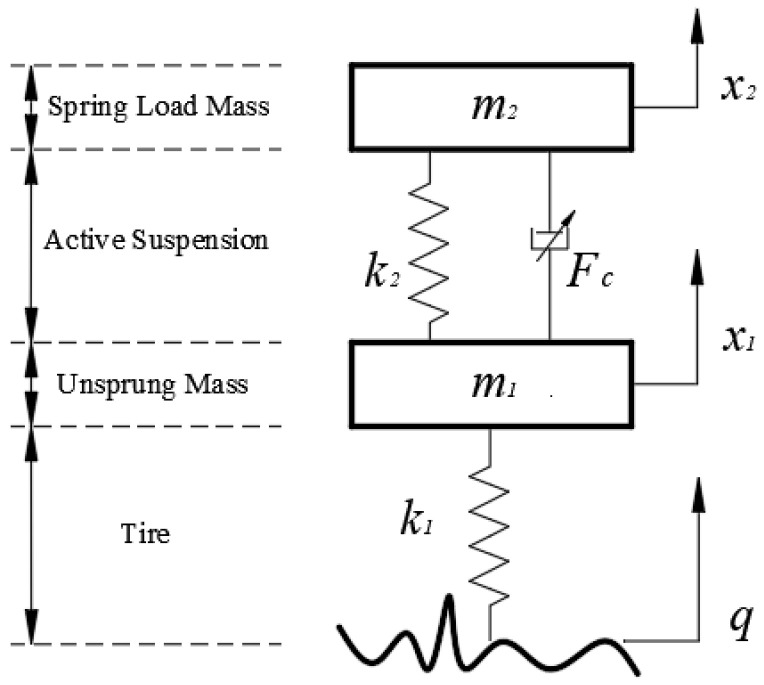
Fractional-order nonlinear active suspension system.

In this paper, a fractional-order nonlinear suspension system model is constructed mainly based on the viscoelastic properties of the employed adjustable-damped oil–air actuator. The constructed active model of the 1/4 vehicle suspension system [28] is shown in Figure 3 below. In this figure, $F_{k}$ denotes the elastic force output from the nonlinear elastic element, $m_{2}$ represents the sprung load mass, $m_{1}$ represents the unsprung load mass, $k_{1}$ is the tire stiffness, and *q* represents the road excitation.

The equation for the nonlinear elastic force $F_{k}$ in the figure is shown in the following equation.

(5) $$F_{k}=k_{2}(x_{2}-x_{1})+e{\left(x_{2}-x_{1}\right)}^{3}$$

where $k_{2}$ is the linear stiffness of the elastic element and *e* is the nonlinear coefficient of the elastic element.

From Figure 3 combined with Equation (5), the differential equation of motion of the suspension system can be obtained as follows:

(6) $$\left\{ \begin{array}{l} m_{2}{\ddot{x}}_{2}+k_{2}(x_{2}-x_{1})+e{\left(x_{2}-x_{1}\right)}^{3}\\ +b\cdot c\cdot D^{p}(x_{2}-x_{1})=0\\ m_{1}{\ddot{x}}_{1}-k_{2}(x_{2}-x_{1})-e{\left(x_{2}-x_{1}\right)}^{3}\\ -b\cdot c\cdot D^{p}(x_{2}-x_{1})+k_{1}(x_{1}-q)=0 \end{array}\right.$$

In the study of the ¼ suspension system, the conventional passive nonlinear suspension system model differential equation of motion is shown in the following equation.

(7) $$\left\{ \begin{array}{l} m_{2}{\ddot{x}}_{2}+k_{2}(x_{2}-x_{1})+e{\left(x_{2}-x_{1}\right)}^{3}\\ +c\cdot ({\dot{x}}_{2}-{\dot{x}}_{1})=0\\ m_{1}{\ddot{x}}_{1}-k_{2}(x_{2}-x_{1})-e{\left(x_{2}-x_{1}\right)}^{3}\\ -c\cdot ({\dot{x}}_{2}-{\dot{x}}_{1})+k_{1}(x_{1}-q)=0 \end{array}\right.$$

where $m_{2}$ denotes the sprung mass; $m_{1}$ denotes the unsprung mass; $k_{1}$ denotes the simulated tire stiffness; *q* denotes the road excitation; $k_{2}$ denotes the linear stiffness of the elastic element; and *e* denotes the nonlinear coefficient of the elastic element.

### 2.4. Pavement Excitation Model

A filtered white noise pavement model was used to construct the pavement input excitation *q* for the active control study process [29], with the following principle formula.

(8) $$\dot{q}(t)=2\pi n_{0}\sqrt{G_{0}v}\cdot w(t)-2\pi f_{0}q(t)$$

where $n_{0}$ is the reference spatial frequency, $n_{0}$ = 0.1 m^−1^; $G_{0}$ is the road surface unevenness coefficient; $f_{0}$ is the space under the cut-off frequency, $f_{0}$ = 0.1 Hz; v is the vehicle speed of 50 km/h. Since the amplitude range of the shaker in the experimental equipment is 0–0.01 m, w(t) is taken as 0.04 unit intensity of Gaussian white noise.

In this paper, regarding the parameters of the international standard ISO 8608 [30] for A, B, and C pavements, the three classes of pavements are combined to form the pavement excitation used in the study, and to consider only the effects caused by pavement unevenness, the pavement excitation formula is designed as shown in the following equation.

(9) $$\dot{q}(t)=\left\{ \begin{array}{l} 2\pi n_{0}\sqrt{G_{1}v}\cdot w(t)-2\pi f_{0}q(t),0\leq t<5\\ 2\pi n_{0}\sqrt{G_{2}v}\cdot w(t-3)-2\pi f_{0}q(t-3),5\leq t<10\\ 2\pi n_{0}\sqrt{G_{3}v}\cdot w(t-6)-2\pi f_{0}q(t-6),10\leq t\leq 15 \end{array}\right.$$

**Figure 4:**
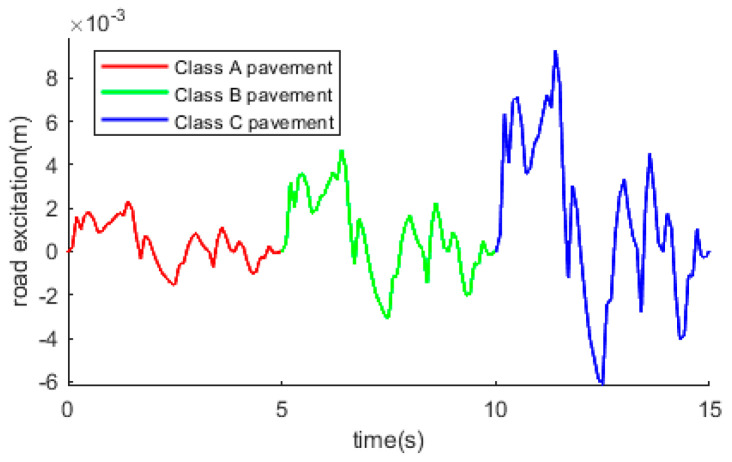
Pavement excitation model.

$G_{1}$, $G_{2}$, and $G_{3}$ are the geometric means of pavement unevenness coefficients for A, B, and C class pavements, with values of 1.6 × $10^{-5}\;m^{3}$, 6.4 × $10^{-5}\;m^{3}$, and 2.56 × $10^{-4}\;m^{3}$, respectively. The pavement excitation images are shown in Figure 4 below.

### 2.5. Electric Oil–Pneumatic Actuator Model

The oil and gas–electric actuator [31] mainly consists of a DC motor, a worm gear reducer, and an oil and gas damper with adjustable damping. The reasons for connecting the worm gear reducer to the DC motor and the oil and gas damper are as follows:

(1) Improvement of motor position control accuracy: Due to the small rotation angle of the motor shaft, its position control accuracy is relatively low. In addition, dampers usually require a smaller adjustment range. Therefore, installing a gear reducer helps to ensure high accuracy of motor position control.
(2) Increased torque: In an isometric drive, the output torque of the motor may not be sufficient to drive the rotation of the damper adjustment knob. By installing a gear reducer, the output torque can be effectively increased to ensure proper operation of the damper.
(3) Reduced space occupancy: The turbine worm gear reducer is smaller in size compared to the gear reducer, which helps to reduce the space occupied by the actuator and improve the compactness of the overall system.

**Figure 5:**
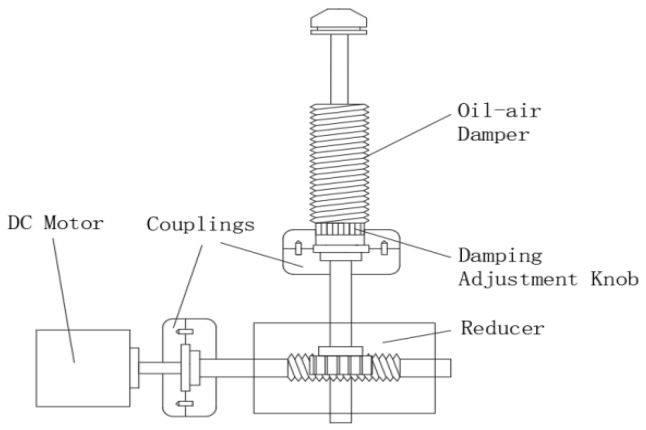
Mechanical structure diagram of the electric oil–pneumatic actuator.

The simple mechanical structure of an electric oil and gas actuator is shown in Figure 5.

The known worm gear reducer reduction ratio is *i*, when the motor angle position is 0 (motor shaft rotation 0 rad), and the scale parameter *b* = 1 in Equation (2). When the motor angular position reaches the maximum value of damping adjustment (when the motor shaft rotates 2π/*i* rad), the scale parameter *b* = 10, from which the relationship between the motor shaft rotation angle θ and the parameter *b* can be obtained, as shown in the following equation.

(10) $$b=\frac{9i\theta +2\pi }{2\pi }$$

The relationship between the output damping force of the damper and the rotation angle of the motor shaft can be obtained by combining Equations (4) and (10):

(11) $$F_{c}=(\frac{9i\theta +2\pi }{2\pi })\cdot c\cdot D^{p}(x_{2}-x_{1})$$

The above equation can be used to control the output damping force of the actuator by controlling the angle of rotation of the motor.

### 2.6. DC Motor Mathematical Model

**Figure 6:**
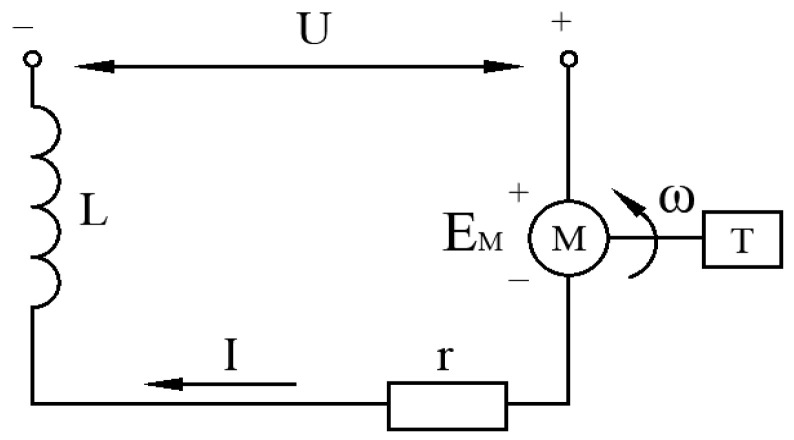
DC motor-equivalent circuit diagram.

The equivalent circuit diagram of the DC motor is shown in Figure 6 below. In the figure, *U* represents the supply voltage, *L* is the motor inductance, *I* is the armature circuit current, *R* is the motor internal resistance, $E_{M}$ is the motor-induced electric potential, *ω* is the motor shaft rotation angular velocity, and *T* is the motor output torque.

From Figure 6 above, the motor voltage balance equation can be obtained as shown in the following equation.

(12) $$U=Ir+L\frac{dI}{dt}+E_{M}$$

where *t* is the time.

The equations of induction potential, electromagnetic torque, and torque balance of a DC motor are known, as shown in Equations (13), (14) and (15), respectively.

(13) $$E_{M}=K_{E}\cdot \omega$$

(14) $$T=K_{T}\cdot I$$

(15) $$T=J\frac{d\omega }{dt}+T_{d}$$

where $K_{E}$ is the counter-electromotive force constant, $K_{T}$ is the torque constant, *J* is the total rotational inertia of the working mechanical system converted to the motor shaft, and $T_{d}$ is the load torque. It is known that the angular velocity of motor shaft rotation *ω* = d*θ*/d*t*, and the mathematical model equation of the DC motor can be obtained from the above Equations (12)–(15).

(16) $$\left\{ \begin{array}{l} \frac{dI}{dt}=(U-Ir-K_{M}\cdot \frac{d\theta }{dt})/L\\ \frac{d^{2}\theta }{dt^{2}}=(K_{T}\cdot I-T_{d})/J \end{array}\right.$$

For the Laplace transform of the above Equation (16), due to the effect of the reducer, the load torque of the motor is very small and can be neglected here, and the result of the transformation is shown in the following equation.

(17) $$\left\{ \begin{array}{l} L\cdot I(s)\cdot s=U(s)-I(s)r-K_{M}\cdot s\cdot \theta (s)\\ J\cdot s^{2}\cdot \theta (s)=K_{T}\cdot I(s) \end{array}\right.$$

where s is a complex parametric variable. For the derivation of the mathematical–physical model of the DC motor here, all parameter values are assumed to be accurately identified at the theoretical level.

From the above equation, the transfer function *H*(*s*) of the DC motor turning angle θ and the motor voltage *U* is given by the following equation.

(18) $$H(s)=\frac{\theta (s)}{U(s)}=\frac{K_{T}}{JL\cdot s^{3}+Jr\cdot s^{2}+K_{M}K_{T}\cdot s}$$

## 3. Principle of Active Control of Suspension System

**Figure 7:**
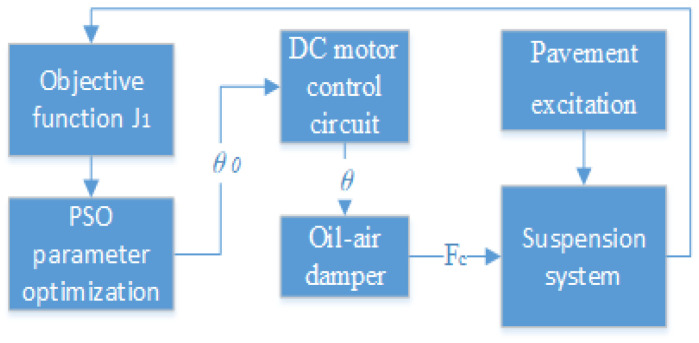
Control schematic of the suspension system.

**Figure 8:**
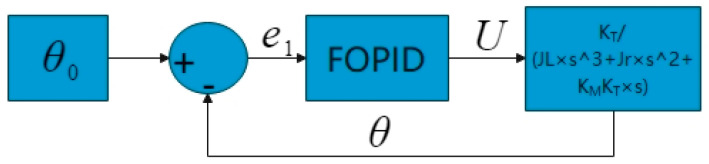
DC motor control circuit schematic.

In this study, the objective function of the suspension system is optimized by using the particle swarm algorithm so that the controller outputs the ideal rotation angle parameter of the motor, and the control principle is schematically shown in Figure 7. The particle swarm algorithm is used to optimize the simple harmonic excitation objective function $J_{1}$, with different frequencies and amplitudes to derive the parameter of the ideal rotation angle $\theta_{0}$. The DC motor control circuit outputs the actual rotation angle *θ* according to the ideal rotation angle $\theta_{0}$, and the oil–air damper adjusts the damping according to the actual rotation angle output by the motor, to realize the output of the damping force $F_{c}$ to achieve a superior suspension control effect. In Figure 7, the working principle of the DC motor control circuit is mainly demonstrated. As shown in Figure 8, the current DC motor rotation angle *θ* forms a deviation value $e_{1}$ from the given rotation angle $\theta_{0}$, and the output power supply voltage *U* is controlled by the FOPID control algorithm, thus realizing the precise control of the DC motor rotation angle.

### 3.1. FOPID Controller Simulation Design

The transfer function of the FOPID controller is shown in the following equation.

(19) $$G(s)=K_{p}+K_{i}/s^{-\lambda }+K_{d}s^{\mu }$$

In this paper, a modified Oustaloup filter [32] is used to implement the approximation of the fractional-order calculus operator $s^{\alpha }$. The main steps in constructing the fractional-order calculus operator are as follows:

(1) Determine the filter order *N* and the approximate frequency band [$w_{b}$, $w_{h}$].
(2) Calculate the zero poles $w_{k}^{\prime }$ and $w_{k}$, which are calculated as shown in Equations (20) and (21).
  (20) $$w_{k}^{\prime }=w_{b}w_{\mu }^{(2k-1-\alpha )/N},w_{k}=w_{b}w_{\mu }^{(2k-1+\alpha )/N}$$
  (21) $$k=w_{h}^{\alpha },w_{\mu }=\sqrt{w_{h}/w_{b}}$$
(3) Finally, the approximation to the fractional-order calculus operator is completed, as shown in Equation (22).
  (22) $$s^{\alpha }={\left(\frac{dw_{h}}{b}\right)}^{\alpha }(\frac{ds^{2}+bw_{h}s}{d(1-\alpha )s^{2}+bw_{h}s+d\alpha })\prod_{k=1}^{N}\frac{s+w_{k}^{\prime }}{s+w_{k}}$$

The above equation *α* must be satisfied (0 < *α* < 1). In general, the approximate frequency band is set as [0.001, 1000], the filter order *N* is 5, and the weighting parameters are selected as *b* = 10, *d* = 9 to meet the accuracy requirements. The improved Oustaloup filter for $s^{-\lambda }$ and $s^{-\mu }$ that approximates the fractional-order calculus operator is designed from Equation (35) above.

### 3.2. Numerical Implementation of FOPID Controller

The FOPID controller in the experimental control circuit needs to be designed using a discretized FOPID formulation, and in this paper, we use the Grünwald–Letnikov formulation, whose differential definition is shown in Equation (2). The control law of FOPID is shown in the following equation [33,34,35].

(23) U(t)=Kpe1(t)+Ki·Dt−λt0e1(t)+Kd·Dtμt0e1(t)

where Dt0t−λ and Dt0tμ are fractional-order calculus operators, where *λ* and *μ* must be real numbers, *t* is the independent variable, and $t_{0}$ is the lower bound of the variable, where the independent variable *t* is time. The uniform fractional-order calculus operator Dt0tα is defined as follows:

(24) Dtαt0f(t)={∫t0tf(τ)dτ−α,α<0f(t),α=0dαdtαf(t),α>0

where *α* is the fractional order.

The conjunction (2) and (23) gives:

(25) $$U(t)=K_{p}e(t)+K_{i}\underset{h\to 0}{\lim}\frac{1}{h^{-\lambda }}\sum_{j=0}^{\left[(t-t_{0})/h\right]}c_{j}e(t-jh)+K_{d}\underset{h\to 0}{\lim}\frac{1}{h^{\mu }}\sum_{j=0}^{\left[(t-t_{0})/h\right]}d_{j}e(t-jh),(\lambda ,\mu >0)$$

where $c_{j}$ and $d_{j}$ are the integral term coefficients and differential term coefficients, respectively. The fractional-order calculus equation is generally implemented by numerical approximation. The two coefficients can be approximated by using the following recursive equations.

(26) $$c_{j}=[1-(1-\lambda )/j]c_{j-1},c_{0}=1,j=1,2,3\cdots$$

(27) $$d_{j}=[1-(1+\mu )/j]d_{j-1},d_{0}=1,j=1,2,3\cdots$$

When the calculation step h chosen in the above equation is small enough, the limit-finding operation in the above equation can be ignored, and $t_{0}$ = 0 in this paper, the following equation is obtained.

(28) $$U(t)=K_{p}e(t)+K_{i}h^{\lambda }\sum_{j=0}^{\left[t/h\right]}c_{j}e(t-jh)+K_{d}h^{-\mu }\sum_{j=0}^{\left[t/h\right]}d_{j}e(t-jh),(\lambda ,\mu >0)$$

From the above equation, the discretization equation for FOPID can be obtained as follows:

(29) $$U_{k}=K_{p}e_{k}+K_{i}h^{\lambda }\sum_{j=0}^{k}c_{j}e_{k-j}+K_{d}h^{-\mu }\sum_{j=0}^{k}d_{j}e_{k-j},(\lambda ,\mu >0),(k=1,2,3\cdots )$$

To describe the FOPID control circuit more clearly, the flow of FOPID position control is described in Algorithm 1.
**Algorithm 1:** FOPID position control flow.FOPID position control algorithmInput: Control circuit controller parameters $k_{p}$, $k_{i}$, $k_{d}$, *λ*, *μ* ideal angle of rotation $\theta_{0}$.Output: actual rotation angle *θ*.For (*j* = 0; *j* < *k*; *j*++); doCalculate the error between the target value and the actual value: $e_{k}=\theta_{0}-\theta$.Calculate the binomial coefficients $c_{j}$ and $d_{j}$ from Equations (26) and (27).From Equation (29) calculate the voltage of the input motor *U*.Error transfer: $e_{k-j-1}=e_{k-j}$Return θ.

### 3.3. Judgment Condition of Damping Adjustment

In this paper, the pavement unevenness of the input pavement excitation is mainly used as the basis for judging the damping adjustment. Based on the group’s previous research on pavement unevenness [36], the pavement identification method will not be repeated here. The known classification criteria for pavement unevenness are shown in Table 1.

According to the data in Table 1, the judgment basis for designing the damping adjustment is shown in the following equation:

(30) $$c_{s}=b\cdot c=\left\{ \begin{array}{l} \frac{9i\theta_{\left[q(G_{1})\right]}+2\pi }{2\pi }\cdot c,8\leq G_{0}\leq 32\\ \frac{9i\theta_{\left[q(G_{2})\right]}+2\pi }{2\pi }\cdot c,32<G_{0}<128\\ \frac{9i\theta_{\left[q(G_{3})\right]}+2\pi }{2\pi }\cdot c,128<G_{0}<512 \end{array}\right.$$

According to Equation (30), the total damping coefficient $c_{s}$ of the suspension system is the optimal damping coefficient obtained by parameter search when the road surface unevenness is $G_{1}$, which corresponds to level A of the road surface. For the road surface unevenness $G_{2}$, the total damping coefficient $c_{s}$ of the suspension system is the best damping coefficient obtained by parameter optimization, which corresponds to level B of the road surface. In case of $G_{3}$, the total damping coefficient $c_{s}$ is the best damping coefficient obtained by parameter optimization, which corresponds to the suspension system at level C. The damping coefficient $c_{s}$ is the best damping coefficient obtained by parameter optimization. This means that the damping coefficient can be flexibly adjusted according to changes in road smoothness, allowing the suspension system to achieve a more stable damping effect under all road conditions.

### 3.4. Objective Function and Constraints

Before determining the parameters to be searched, the objective function of the search and the constraints to be satisfied by the search results need to be specified. Suspension system performance is evaluated based on body acceleration, suspension dynamic deflection, and tire dynamic loads, which interact with each other. Body acceleration is used to evaluate ride smoothness, tire dynamic loads are used to evaluate handling stability, and suspension dynamic deflection measures the effect on body attitude. Therefore, when performing active suspension control, handling stability needs to be taken into account and the suspension dynamic deflection needs to be controlled within an acceptable range based on optimizing the vehicle ride smoothness. Therefore, the objective function $J_{1}$ is constructed as shown below.

(31) $$J_{1}=\rho_{1}\frac{rms({\ddot{x}}_{2})}{rms{\left({\ddot{x}}_{2}\right)}_{p}}+\rho_{2}\frac{rms(x_{2}-x_{1})}{rms{\left(x_{2}-x_{1}\right)}_{p}}+\rho_{3}\frac{rms[k_{1}(x_{1}-q)]}{rms{\left[k_{1}(x_{1}-q)\right]}_{p}}$$

Meet the constraints:

(32) $$s.t.=\left\{ \begin{array}{l} \frac{rms({\ddot{x}}_{2})}{rms{\left({\ddot{x}}_{2}\right)}_{p}}<1\\ x_{2}-x_{1}<b_{1}\\ \frac{rms[k_{1}(x_{1}-q)]}{rms{\left[k_{1}(x_{1}-q)\right]}_{p}}\leq 1 \end{array}\right.$$

where the subscript *p* represents the passive suspension indicators and rms denotes the root mean square value of each indicator. The purpose of this design is to dimensionless-size each performance index and facilitate the selection of weighting factors. $\rho_{1},\rho_{2},\;\mathrm{and}\;\rho_{3}$ are the weighting coefficients due to the different importance of each index in the suspension system. Let $\sum_{i=1}^{3}\rho_{i}=1$ and determine the weighting coefficient of the required optimization indexes according to the required order of optimization of each index combined with the constraints. Since the permitted travel of the oil and gas damper is 0.04 m in the equiproportional model used in the experiment, $b_{1}=0.04$ is taken in the constraint. When the output of the suspension system does not satisfy the constraint, the weighting coefficients $\rho_{1},\rho_{2},\;\mathrm{and}\;\rho_{3}$ to be readjusted.

### 3.5. Parametric Optimization Principle and Process

In this paper, the particle swarm algorithm [37] is used to find the optimal objective function $J_{1}$ and the goal is to find the optimal turning angle $\theta_{0}$, to achieve the optimal control of body acceleration and improve the smoothness of the vehicle. The principle of the particle swarm algorithm for finding the optimum is shown below.

The initial parameters of the particle swarm are set, including the inertia factor *w*, acceleration constants $o_{1}$ and $o_{2}$, the number of particles in the swarm *S*, the maximum number of particle iterations *T*, the upper bound $U_{B}$, and the lower bound $U_{L}$ for the parameter search. The position information of particle m is $X_{m}(x_{m1}$, …, $x_{\mathrm{mn}})$. The particle search velocity is $V_{m}(v_{m1}$, …, $v_{\mathrm{mn}}$), *n* is the number of optimization-seeking parameters, i.e., the dimension of the solution space, and *n* = 1 when the optimization is sought for the objective function $J_{1}$. To prevent the particle velocity from being too large and exceeding the set boundary, the velocity of particle m is limited to $-v_{\max}\leq v_{\mathrm{mn}}\leq v_{\max}$, and the part exceeding is taken as the boundary value.

*t* denotes the number of current iterations of the particle, and the particle velocity update formula is shown in the following equations:

(33) $$H(t)=wv_{mn}(t)+o_{1}\cdot r_{1}\cdot [pbest_{mn}(t)-x_{mn}(t)]+o_{2}\cdot r_{2}\cdot [gbest_{n}(t)-x_{mn}(t)]$$

(34) $$v_{mn}(t+1)=\left\{ \begin{array}{l} H(t),-v_{\max}\leq H(t)\leq v_{\max}\\ v_{\max},H(t)>v_{\max}\\ -v_{\max},H(t)<v_{\max} \end{array}\right.$$

The particle position update formula is shown in the following equation:

(35) $$x_{mn}(t+1)=x_{mn}(t)+v_{mn}(t+1)$$

where $\mathrm{pbes}t_{\mathrm{mn}}\left(t\right)$ denotes the particle m individual current optimal position parameter and $\mathrm{gbes}t_{n}\left(t\right)$ denotes the particle swarm global current optimal position parameter. $r_{1}$, $r_{2}$ denote the random number between [0, 1].

The individual m optimal position update equation is

(36) $$pbest_{m}=\left\{ \begin{array}{l} pbest_{m}(t),J[X_{m}(t+1)]\geq J[pbest_{m}(t)]\\ X_{m}(t+1),J[X_{m}(t+1)]<J[pbest_{m}(t)] \end{array}\right.$$

**Figure 9:**
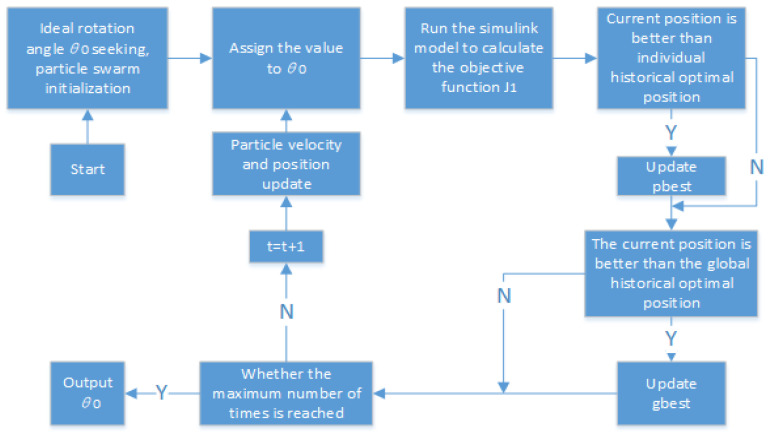
Suspension system parameter optimization flow chart.

The global optimal position update formula is

(37) $$J[gbest(t)]=\min\left\{J[pbest_{1}(t)],\cdots ,J[pbest_{S}(t)]\right\}$$

when the objective function $J_{1}$ is optimized, the upper bound $U_{B}$ is set to [2π/*i*] and the lower bound $U_{L}$ is set to [0], because the maximum rotation angle of the adjustable damping knob is 2π. The rest of the initial parameters are shown in Table 2. The algorithm optimization flow is shown in Figure 9.

After preliminary simulations and experimental studies, combined with the actual parameters of the experimental rig, the values of each parameter can be obtained as shown in Table 3 below.

Among them, the selection of the reduction ratio *i* of the reducer mainly considers the following factors.

As mentioned earlier, if the reduction ratio of the selected reducer is too large, the output torque may not be sufficient to push the damping adjustment knob, resulting in a decrease in control accuracy. Conversely, if the reduction ratio is too small, this will cause the time required for damping adjustment to increase to a given value, thereby slowing down the response of the system. Therefore, the reduction ratio *I* = 1:20 is selected to balance the system performance, taking into account the control accuracy and output torque, while referring to the available reducer models on the market.

**Figure 10:**
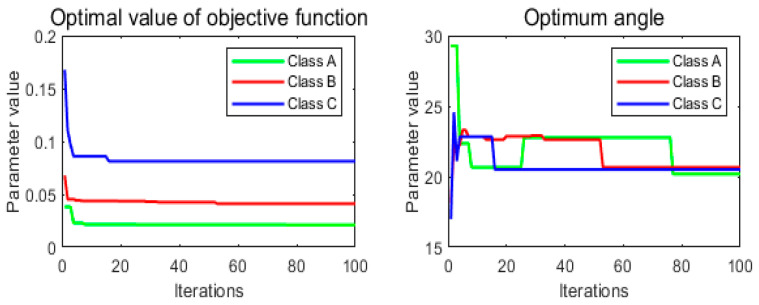
Parametric optimization search results chart.

The simulation model of the suspe”Iio’ systeI is established according to the parameters in Table 3, and then the objective function $J_{1}$ and the ideal turning angle $\theta_{0}$ are searched for under each road level, respectively, and the searched results are shown in Figure 10 below.

From Figure 10, it can be seen that the optimal values of the objective function $J_{1}$ gradually increase with the increase of the pavement grade, which are 0.0211, 0.0453, and 0.0813, respectively, while the optimal values of the ideal turning angle first increase and then decrease, which are 20.1804, 20.6578, and 20.5130, respectively. Compared with the passive suspension, the damping value can be adjusted in time by actively adjusting the rotation angle of the semiactive suspension under different road surfaces. Reference [38] results show that the semiactive suspension under different road surfaces can improve the automotive system dynamics parameter body acceleration by 29.7% compared to the passive suspension. For which the suspension is referred to the parameters of the Ford Granada sedan, the parameters of the suspension system are taken as shown in Table 3. After determining the optimal value of the ideal corner, it is taken as the given value of the controller. The PID algorithm and FOPID algorithm are used for motor corner control, and the control principle is shown in Figure 8. It should be noted that, whether it is PID or FOPID, due to its weighting factor and suspension control, feed energy involved in too many parameters, this paper did not derive the corresponding controller parameters’ optimal functional relationship, only through the references and a large number of simulation and experimental trial cobbled together, to obtain the recommended controller parameters as shown in Table 4.

## 4. Simulation and Experimental Results Study Analysis

**Figure 11:**
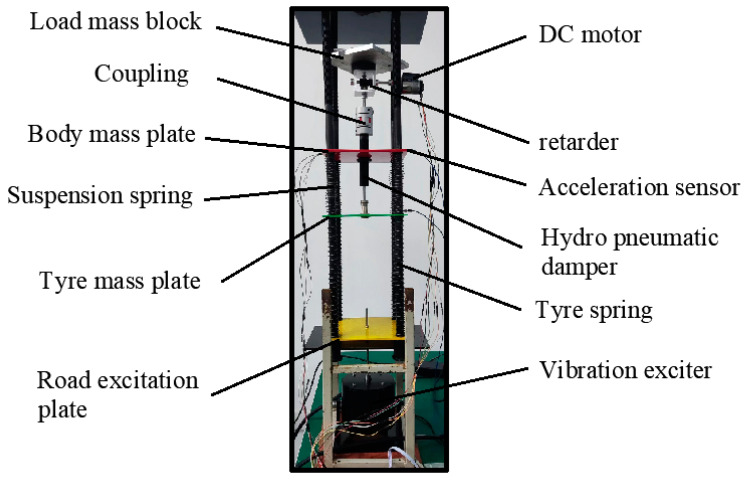
Equivalent proportional oil–pneumatic suspension equipment.

To verify the accuracy of the established fractional-order nonlinear suspension system describing the oil–pneumatic suspension, the superiority of the FOPID controller compared with the PID controller, and the feasibility of the designed electric oil–pneumatic active suspension, simulations, and bench tests were performed for the equiproportional electric oil–pneumatic active suspension device shown in Figure 11.

### 4.1. Study on the Superiority of Fractional-Order Nonlinear Passive Suspension Model

**Figure 12:**
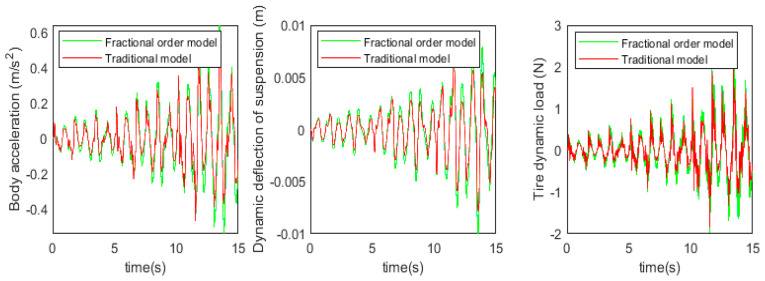
Passive suspension model simulation results.

**Figure 13:**
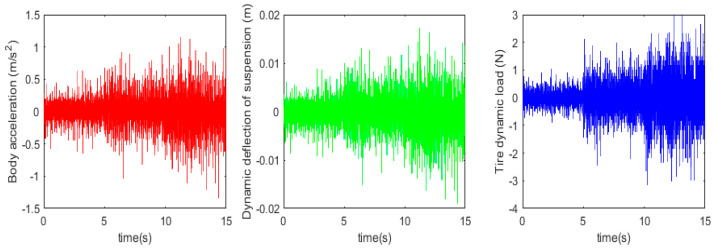
Passive suspension model test results.

Before proceeding to the active suspension control study, the accuracy of the used fractional-order nonlinear passive suspension model is demonstrated. Usually, passive suspension models assume that damping is a linear fixed value, while models that consider fractional-order nonlinear characteristics should be closer to the true value. At this point, *b* = 1, the body acceleration, suspension dynamic deflection, and tire dynamic load under simulation and test were obtained, as shown in Figure 12 and Figure 13 below.

Based on the simulation and test results, the root mean square values of each suspension index were obtained, as shown in Table 5 below.

It can be observed from the data in the above figure and table that the simulation results of the fractional-order nonlinear suspension system model used in this paper are closer to the actual values compared to the conventional nonlinear suspension system model. Specifically, the accuracy of body acceleration is improved by 18.5%, the accuracy of suspension dynamic deflection is improved by 20%, and the accuracy of tire dynamic load is improved by 19.6%. It can be seen that the use of a fractional-order nonlinear suspension system model to simulate the oil and gas suspension system can reflect the actual situation more accurately than the traditional nonlinear model.

### 4.2. FOPID Controller Superiority Verification

**Figure 14:**
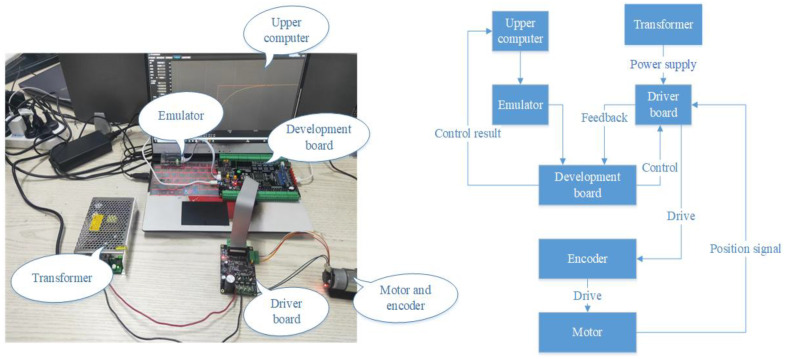
Motor angle control test bench and control flow.

To optimize motor corner control, this paper introduces the FOPID control algorithm to replace the traditional PID control algorithm and verifies the excellent performance of the FOPID control algorithm in motor corner control through simulation and test results. In the actual implementation, this paper chooses the STM32–F407 development board as the carrier of the control algorithm and uses Keil uVision5 to write PID and FOPID control programs in the upper computer and set the initial parameters of the controller. The control driver board is powered by a transformer, which drives the motor through an encoder. The Hall sensor on the driver board is responsible for acquiring the position signal of the motor and feeding it back to the development board. The development board then controls the angle of the motor in real time and feeds the control results to the host computer for display. Figure 14 shows the information about the test equipment and the test flow.

**Figure 15:**
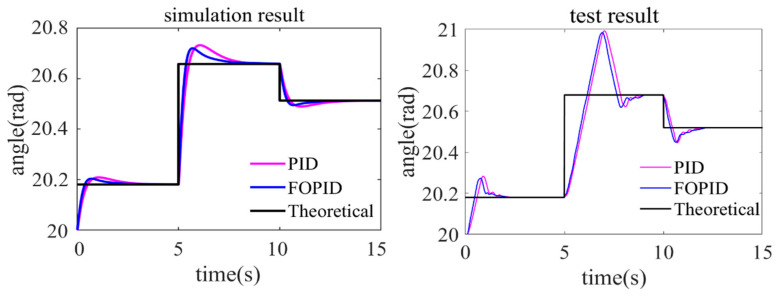
Motor angle control results.

To avoid system instability caused by excessive overshoot, the initial rotation angle is set to 20 rad. The simulation and test results are shown in Figure 15 below.

As shown in the figure, both the simulation result and test result indicate that the FOPID control represented by the blue curve achieves the desired motor angle faster than the PID control represented by the purple curve. In the simulation result, when facing sudden changes in angle, the overshoot of the simulation curve is small, and steady state is achieved within 2.5–3 s. However, in the test result, there is significant overshoot and oscillation, especially during the 5-s angle adjustment with a large step change, which results in a long time to achieve steady state. Nevertheless, compared to the traditional PID controller, the FOPID controller exhibits faster response, smaller overshoot, and shorter steady state time in controlling motor angle.

To provide a more intuitive analysis, the curves in Figure 15 were quantified by calculating the root-mean-square error (RMSE) values between the actual motor angle and the theoretical motor angle under PID and FOPID control. In the simulation, the RMSE for PID control was 0.2629, while the RMSE for FOPID control was 0.2563, resulting in a 2.58% decrease in RMSE. In the experiment, the RMSE for PID control was 0.3158, while the RMSE for FOPID control was 0.2998, representing a 5.34% decrease in RMSE. Furthermore, when comparing simulation and experimental results, the RMSE under PID and FOPID control in the simulation was, respectively, 16.75% and 14.51% smaller than that in the experiment. This could be due to external interference or errors affecting software control in practical applications, or factors such as processing and assembly errors and delayed mechanical responses among parts. However, overall, FOPID control effectively optimizes motor angle control and rapidly improves actuator stability.

**Figure 16:**
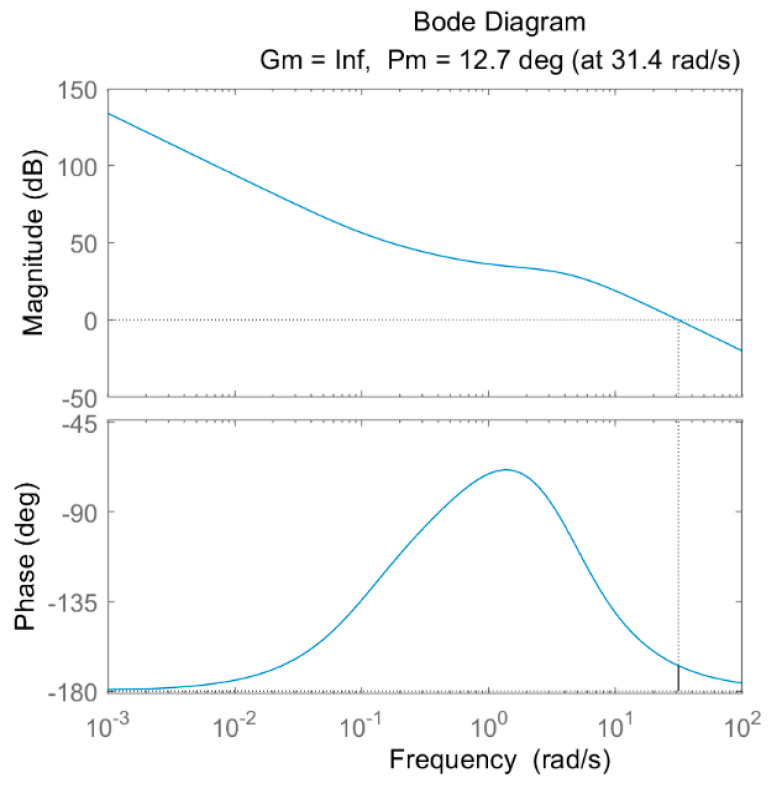
Bode diagram of PID controller.

**Figure 17:**
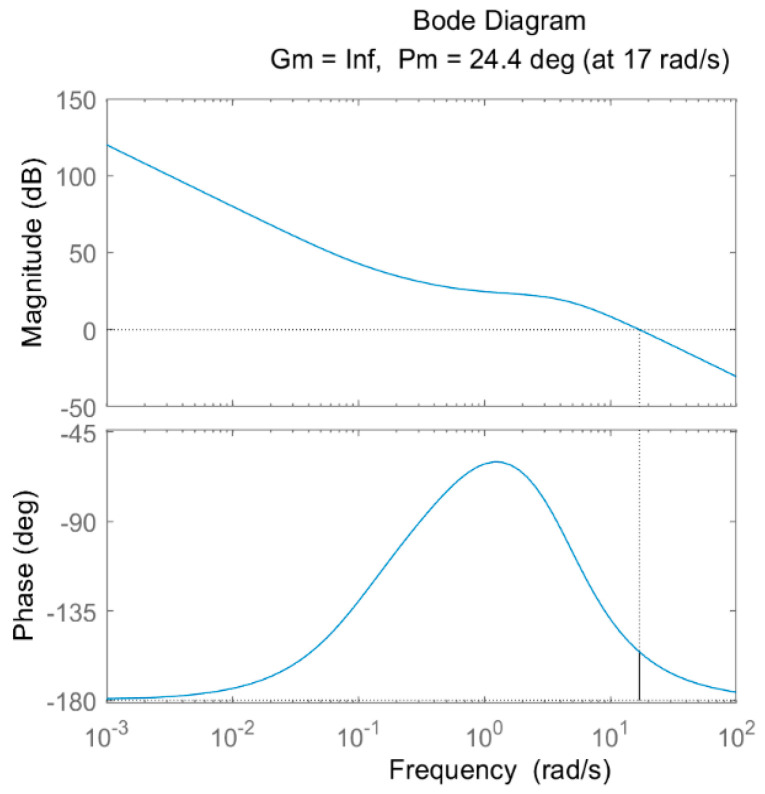
Bode diagram of FOPID controller.

To address the above problem of error between simulation and test, the stability of the system when the system is subjected to external disturbances and errors is analyzed by plotting the Bode plots of the controllers. According to the flowchart and transfer function equation in Figure 8, the values in Table 3 and Table 4 are brought into the solution to obtain the Bode plots of the PID and FOPID controllers, as shown in Figure 16 and Figure 17.

In the Bode plot shown in Figure 16 and Figure 17, the blue line in the Magnitude plot represents the Amplitude curve, and the dash line indicates the coordinate lines for amplitude and frequency at 0. In the Phase plot, the blue line represents the Phase curve, and the dash line indicates the coordinate line for frequency at 0. The phase margin γ in the plot is the phase difference between the corresponding phase–frequency response curve and −180 degrees when the open-loop gain of the system is 0 db (traversing frequency $\omega_{c}$), which is used to evaluate the stability of the system, and the larger the phase margin is, the better the system stability is. According to Figure 16, the PID controller achieves a phase margin $\gamma_{1}$ of 12.7° at a traversal frequency $\omega_{c1}$ of 31.4 rad/s, whereas the FOPID controller achieves a phase margin $\gamma_{2}$ of 24.4° at a traversal frequency $\omega_{c2}$ of 17 rad/s, as shown in Figure 17. Obviously $\gamma_{2}$ > $\gamma_{1}$, which indicates that the FOPID control is a more stable system compared to the conventional PID control.

### 4.3. Suspension Active Control Results Analysis

**Figure 18:**
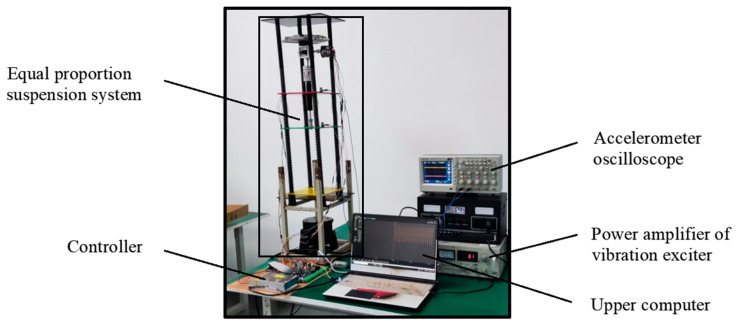
Suspension system test bench.

To verify the feasibility of the designed FOPID position feedback control in an electric-hydrocarbon active suspension system, the body acceleration, suspension dynamic deflection, and tire dynamic loads in the passive and active control states were simulated and experimentally investigated. The constructed test rig for the active suspension system is shown in Figure 18 below. This study aims to evaluate the performance of FOPID control in a real active suspension system to gain a more comprehensive understanding of its potential for improving vehicle dynamic characteristics.

**Figure 19:**
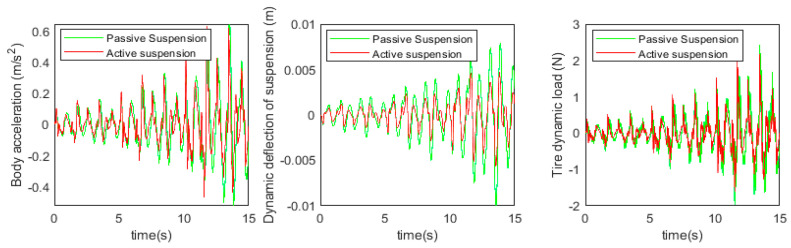
Active and passive suspension simulation results.

**Figure 20:**
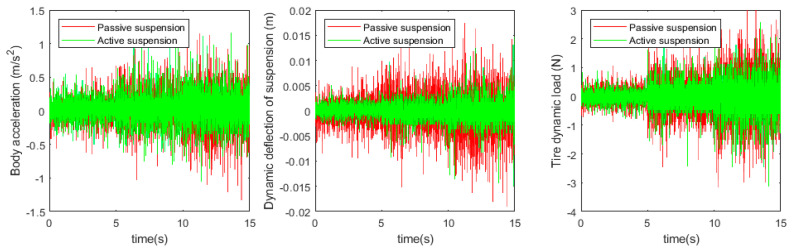
Active and passive suspension test results.

The simulation and experimental results are shown in Figure 19 and Figure 20 below.

Based on the simulation and test results, the root-mean-square values of each suspension index were obtained as shown in Table 6 below.

According to the simulation results, compared with the passive suspension system, the electric oil–air active suspension achieves 75.2% optimization in body acceleration, 57.1% optimization in suspension dynamic deflection, and 75.6% improvement in tire dynamic load. The experimental validation shows that, compared with the traditional passive oil–air suspension system, the designed electric oil–air active suspension can effectively improve the damping performance of the suspension system, which is theoretically feasible. The specific experimental results show that the body acceleration is increased by 89.8%, the suspension dynamic deflection is increased by 56.7%, and the tire dynamic load is increased by 73.4%. This indicates that the electric oil–pneumatic active suspension system has been significantly optimized in terms of body acceleration, suspension dynamic deflection, and tire dynamic load, which verifies the accuracy of the simulation conclusions and confirms the feasibility of the designed structure for engineering applications.

## 5. Conclusions

This study focuses on the electric hydraulic suspension system with nonlinear viscoelastic characteristics to enhance the dynamic performance of the vehicle suspension system. By constructing a model of the electric hydraulic suspension system and optimizing the target parameters of the motor angle, precise control of the motor angle is achieved to improve suspension control force. The study employs FOPID control with a wider range of parameter tuning to achieve higher-precision control of the target parameters. Simulation and experimental validation have confirmed the accuracy and feasibility of this model and approach. The main contributions of this research include the following:

(1) Based on the viscoelastic characteristics of the electric hydraulic suspension and the fractional calculus theory, a fractional-order nonlinear suspension system model is derived. Compared to the simulation results obtained from traditional nonlinear suspension models, the results obtained from the derived model are closer to the experimental values, with an optimization of accuracy by more than 18.5% for all performance indicators.
(2) By using the particle swarm optimization algorithm and considering the road roughness coefficient, the optimization of suspension damping coefficients for different road grades is achieved. The optimized results are then utilized in the digital implementation of the FOPID control circuit for motor angle control. Simulation and experimental validation of the actuator control circuit have confirmed the superiority of FOPID control over traditional PID control.
(3) Through simulation experiments on the fractional-order nonlinear hydraulic suspension system, quantified results demonstrate that FOPID control outperforms traditional suspension systems in terms of vehicle body acceleration, suspension travel, tire load, and other evaluation indicators, leading to improved ride comfort and handling stability.

Plan for further development:

(1) The active control in this study is limited to offline data processing under experimental conditions, and real-time control of the suspension-damping effect is not achieved. Therefore, the next research plan involves real-time optimal control of the suspension system through identification based on methods like least squares or Kalman filtering, cloud data processing, and utilizing hardware such as dSPACE.
(2) During the research process, this study mainly compared the improvement in damping performance of the suspension system between FOPID and traditional PID control. Other control algorithms, such as optimal robust control algorithms, fuzzy PID control algorithms, and genetic algorithm–optimized LQG control algorithms, were not utilized for comparison.

## Figures and Tables

**Table 1:** Road surface unevenness classification standards.

Pavement Grade	$G_{0}(n_{0})/10^{-6}\;m^{3}$		
	Lower Limit	Geometric Mean	Upper Limit
A	8	16	32
B	32	64	128
C	128	256	512

**Table 2:** Initial parameters of the particle swarm algorithm.

Parameters	Value
w	1
$o_{2}$	0.5
T	100
$o_{1}$	0.5
S	10
$v_{\max}$	10

**Table 3:** Suspension system parameters table.

Parameters	Value	Parameters	Value
$m_{1}/\mathrm{kg}$	0.4	$k_{1}/N\cdot m^{-1}$	1680
$m_{2}/\mathrm{kg}$	2.7	$k_{2}/N\cdot m^{-1}$	155
*e*	3	$c/N\cdot s\cdot m^{-1}$	8
*p*	0.85	i	1/20
$\rho_{1}$	0.5	r/Ω	14.4
$\rho_{2}$	0.2	$\rho_{3}$	0.3
L/H	0.3	$J/\mathrm{kg}\cdot m^{2}$	0.02

**Table 4:** Controller parameter selection.

Control Algorithms	Parameter				
	$K_{p}$	$K_{i}$	$K_{d}$	λ	μ
PID	10	1	10	1	1
FOPID	11	1	15	0.8	0.9

**Table 5:** Root mean square values of performance indicators.

Performance Indicators	Numerical Access				
	Test	Traditional Model Simulation	Difference from Test	Fractional-Order Model Simulation	Difference from Test
Body acceleration/m·s^−2^	0.2095	0.1427	31.9%	0.1814	13.4%
Dynamic deflection/m	0.0030	0.0022	26.7%	0.0028	6.7%
Tire dynamic load/N	0.5646	0.4215	25.3%	0.5326	5.7%

**Table 6:** Root-mean-square values of performance indicators.

Performance Indicators	Numerical Access			
	Passive Suspension Simulation	Active Suspension Simulation	Passive Suspension Experiment	Active Suspension Experiment
Body acceleration/m·s^−2^	0.1814	0.1384	0.2095	0.1882
Dynamic deflection/m	0.0028	0.0016	0.0030	0.0017
Tire dynamic load/N	0.5326	0.4025	0.5646	0.4142

## Data Availability

Data are contained within the article.
